# *In Vitro* Cytotoxicity Evaluation of Four Vital Pulp Therapy Materials on L929 Fibroblasts

**DOI:** 10.1155/2014/191068

**Published:** 2014-03-03

**Authors:** Aniket S. Wadajkar, Chul Ahn, Kytai T. Nguyen, Qiang Zhu, Takashi Komabayashi

**Affiliations:** ^1^Department of Bioengineering, University of Texas at Arlington, Arlington, TX 76019, USA; ^2^Joint Biomedical Engineering Program, University of Texas at Arlington and University of Texas Southwestern Medical Center, Dallas, TX 75390, USA; ^3^Department of Clinical Sciences, University of Texas Southwestern Medical Center, Dallas, TX 75390-9066, USA; ^4^Division of Endodontology, University of Connecticut Health Center, Farmington, CT 06030-1715, USA; ^5^Department of Endodontics, West Virginia University School of Dentistry, One Medical Center Drive, P.O. Box 9450, Health Science Center North, Morgantown, WV 26506-9450, USA

## Abstract

The aim of this study was to evaluate cytotoxicity of direct pulp capping materials such as Dycal, Life, ProRoot MTA, and Super-Bond C&B on L929 fibroblasts. Freshly mixed or set materials were prepared and eluted by incubation with cell culture medium for working time period (fresh) or for 6 hours (set). The cells were exposed to media containing elutes for 24 hours, after which the cell survival was evaluated by MTS assays. In freshly mixed materials, average ± standard deviation % cell viabilities were 40.2 ± 14.0%, 43.7 ± 16.0%, 72.9 ± 12.7%, and 66.0 ± 13.6% for Dycal, Life, ProRoot MTA, and Super-Bond C&B, respectively. There was no statistical difference in cell viabilities among material groups, whereas in set materials, the cell viabilities were 48.7 ± 14.8%, 37.2 ± 10.6%, 46.7 ± 15.2%, and 100 ± 21.9% for Dycal, Life, ProRoot MTA, and Super-Bond C&B, respectively. Super-Bond C&B showed more cell viabilities than the other three material groups (P < 0.05). The four vital pulp therapy materials had similar cytotoxicity when the materials were fresh. Super-Bond C&B was less cytotoxic than Dycal, Life, and ProRoot MTA after the materials were set, which suggests the use of SB-C&B in future *in vivo* clinical investigations.

## 1. Introduction

Direct pulp capping is a treatment for exposed vital pulp, which uses a dental material to facilitate both the formation of reparative dentin from odontoblasts [1] and the maintenance of vital pulp [2]. These materials include calcium hydroxide or calcium hydroxide-based cements such as Dycal and Life [3, 4], mineral trioxide aggregate (MTA; ProRoot MTA) [5], and adhesive resins [6]. Selection of pulp capping materials is important to ensure dental pulp cell vitality. Historically, Hermann [7] discovered that calcium hydroxide is effective in repairing a pulp exposure site. Calcium hydroxide possesses antibacterial properties and promotes pulp tissue repair [8]. Thus, it is considered the “gold standard” for direct pulp capping, and it has a long record of clinical success [9].

MTA was developed in 1993 and has been successfully used for pulp capping [10]. When MTA powder is mixed with water, its calcium oxide component reacts with the water and forms calcium hydroxide. Thus, MTA slowly releases calcium hydroxide while setting [11]. In addition, adhesive systems have also been suggested for use as direct pulp capping materials [12]. However, it has been believed that resin systems are inferior to calcium hydroxide-based cements, including MTA [13]. The exception would be a methyl methacrylate-/tributylborane- (MMA/TBB-) based adhesive system, which is known commercially as Super-Bond C&B (SB-C&B) in Japan and C&B Metabond in USA. Feasibility of the MMA/TBB resin as a direct pulp capping material has been suggested from the low cytotoxicity in rat dental pulp cell lines [14]. Evident dentin bridge formation and wound healing were reported to occur in the same manner as calcium hydroxide after application of the resin to exposed pulp surface in animals [15]. Moreover, favorable clinical results were reported in direct pulp capping of the resin [16].

Despite a potential of the resin, very few studies have been conducted to compare biocompatibility of the resin and conventional direct pulp capping materials in fresh (short term before material setting) and set conditions. In this study, we used mouse L929 fibroblasts that are routinely used for cytotoxicity studies in dental materials and have been shown to be more prone to the toxic effects of products than human fibroblasts [17]. The aim of this study, as a part of biocompatibility evaluation of materials, is to evaluate the cytotoxicity of direct pulp capping materials such as Dycal, Life, MTA, and SB-C&B in fresh and set conditions using mouse L929 fibroblasts.

## 2. Materials and Methods

### 2.1. Materials

Pulp capping materials Dycal (Dentsply Caulk, Milford, DE, USA), Life (Kerr, Orange, CA, USA), MTA Gray (Dentsply Tulsa, Tulsa, OK, USA), and SB-C&B (Sun Medical Co., Shiga, Japan) were purchased and used without any modifications. Working times and setting times of these materials were summarized in Table 1. L929 mouse fibroblasts were obtained from American Type Culture Collection (ATCC, Manassas, VA, USA). Cells were grown in Dulbecco's Modified Eagle Medium (DMEM; Invitrogen, Carlsbad, CA, USA) supplemented with 10% fetal bovine serum (Hyclone Laboratories Inc., Logan, UT, USA) and 1% penicillin-streptomycin (Gibco BRL, Gaithersburg, MD, USA) under standard cell culture conditions (37°C and 5% CO_2_).

### 2.2. Methods

The cytotoxicity of the four materials was tested in fresh and set conditions. For fresh conditions, the materials were mixed according to manufacturer's instructions and freshly mixed materials were placed into 24-well plates at 0.1 g/well. One mL DMEM was added to the wells and the plates were incubated in a cell culture incubator for the material working time as specified in Table 1 (Dycal for 3 minutes, Life for 7 minutes, MTA for 6 hours, and SB-C&B for 6 minutes). The DMEM containing elute was removed and used for further cytotoxicity assay. Six wells were used for each material. For set conditions, materials were mixed according to manufacturer's instructions and placed into the 24-well plates at 0.1 g/well. The plates were incubated in the cell culture incubator to allow the materials to completely set. Setting of materials was checked with a dental explorer by confirming no indentation. One mL DMEM was then added to the wells containing the set materials and the plates were incubated again for 6 hours. The DMEM containing elute was removed and used for further cytotoxicity assay. Six wells were used for each material.

For the cytotoxicity assay, L929 fibroblasts were seeded into 96-well plates at 5000 cells/well and incubated for 24 hours to allow cell adhesion. The medium was then replaced by 200 *μ*L of the DMEM containing elute material from the different groups. Cells exposed to DMEM only served as control group. After an incubation of 24 hours, cell viability was evaluated by 3-(4,5-dimethylthiazol-2-yl)-2,5-diphenyltetrazolium bromide (MTS; CellTiter 96 AQ_ueous_ One Solution Cell Proliferation Assay, Promega, Madison, WI, USA) assays according to the manufacturer's instructions. Cell viability was then calculated as percentage of the control group.

### 2.3. Statistical Analysis

ANOVA test was conducted to investigate if there were significant differences in cell viabilities between experimental and control groups, and Student's *t*-tests were conducted to identify which material was significantly different from control with Dunnet's correction. Cell viabilities among material groups were also compared using ANOVA test and Student's *t*-tests with Bonferroni correction.

## 3. Results

**Figure 1:**
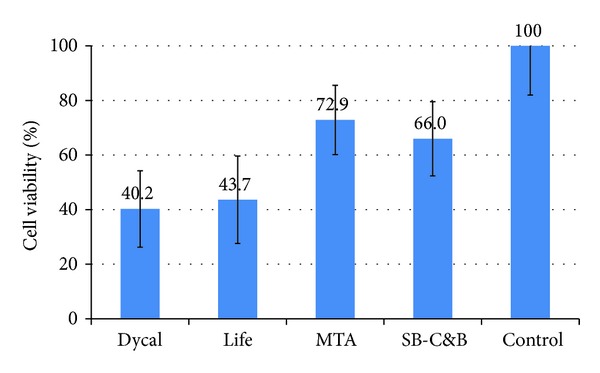
Cell viability of L929 fibroblasts upon exposure to media containing elutes of freshly mixed materials. No significant difference among material groups (*P* < 0.05).

**Figure 2:**
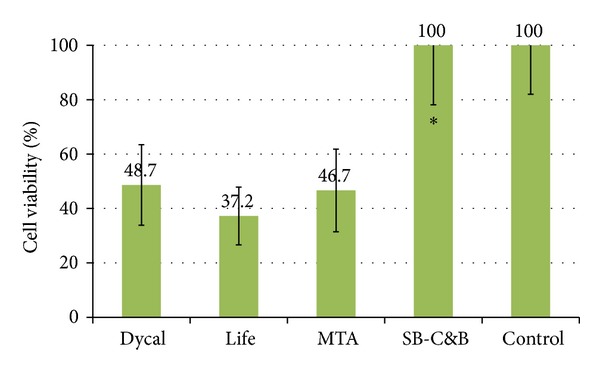
Cell viability of L929 fibroblasts upon exposure to media containing elutes of set materials. *Significantly different from Dycal, Life, and MTA groups (*P* < 0.05).

In freshly mixed materials, average ± standard deviation % cell viabilities were 40.2 ± 14.0%, 43.7 ± 16.0%, 72.9 ± 12.7%, and 66.0 ± 13.6% for Dycal, Life, MTA, and SB-C&B, respectively. There was no statistical difference in cell viabilities among material groups in the fresh conditions (Figure 1). In set materials, average ± standard deviation % cell viabilities were 48.7 ± 14.8%, 37.2 ± 10.6%, 46.7 ± 15.2%, and 100 ± 21.9% for Dycal, Life, MTA, and SB-C&B, respectively (Figure 2). SB-C&B showed more cell survival than the other three materials in the set conditions (*P* < 0.05).

## 4. Discussion

This study evaluated and compared the cytotoxicity of four pulp capping materials that had not been compared in the same study previously. The four materials had similar cytotoxicity levels when freshly mixed. However, after the materials were set, SB-C&B was less cytotoxic than Dycal, Life, and MTA. Previous studies have found that calcium hydroxide had the best clinical outcome for pulp capping [6, 18]. Calcium hydroxide and MTA stimulate the formation of reparative dentin, while no hard tissue barrier was formed adjacent to the adhesive system. Generally, it has been believed that resin systems are inferior to Dycal, based on histology reports of direct pulp capping for mechanically exposed human teeth [6, 13]. The bonding agents, including All Bond 2 [19], Single Bond [6], Clearfil Liner Bond 2 [20], and Scotchbond Multi-Purpose [13], and composite resin (Z100) were applied to the pulp for 2−10 months. As a control, Dycal was used in all these studies. These reports concluded that Dycal was better in formation of reparative dentin and pulp repair than the resin systems. Therefore, by comparison with our studies, we can speculate that the clinical outcome of pulp capping may not be related to the cytotoxicity of the tested pulp capping materials.

Hirschman et al. [21] reported that Dycal cytotoxicity was high, whereas Tani-Ishii et al. [22] reported that Dycal cytotoxicity was low. Our results for freshly mixed Dycal is in agreement with that of Hirschman et al., while our result for set Dycal is in agreement with that of Tani-Ishii et al. Regarding MTA, it was previously reported that the cytotoxicity of MTA is low [23]. These results are in agreement with our result for freshly mixed MTA. According to Yasuda et al., [24] MTA and SB-C&B showed no cytotoxicity, while Dycal indicated high cytotoxicity between 5 and 72 hours. These results differ from our observations; however, the high cytotoxicity of Dycal in freshly mixed form is in agreement with our results. The difference of our results with previous studies is probably due to the different cell lines, material preparation, and test methods.

In set form, SB-C&B group had the highest cell viability among all the material groups tested. SB-C&B consists of methyl-methacrylate (MMA) monomer, polymethylmethacrylate (PMMA) polymer, and Tri-n-butyl borane (TBB) catalyst, which is an initiator for polymerization. Tronstad and Spangberg [25] studied the pulp responses to a composite resin (Concise) and an MMA-TBB-based resin (Polycap) and found the MMA-TBB-based resin had a lower cytotoxicity than the composite resin. No severe response was reported for the MMA-TBB resin. The success of MMA-TBB resins like SB-C&B can be attributed to several material properties, such as low cytotoxicity and high bonding strength. Based on a concentration that inhibited 50% of cell growth, MMA is the least cytotoxic among the monomers used in dentistry [26]. Also, the residual MMA is low after setting. Dycal, Life, and MTA are subject to dissolution and continuous release of calcium hydroxide, which is responsible for the cytotoxicity [27]. Fridland and Rosado [27] showed that the continuous release of calcium hydroxide after setting causes cytotoxicity *in vitro*, but they suggested that it might be beneficial for pulp capping *in vivo*. The calcium hydroxide-based cements and MTA may continue dissolving the proteins for a longer period of time, thus promoting dentinogenesis. In addition, the calcium hydroxide-based cements and MTA possess antibacterial properties, thus preventing bacterial penetration. These are the potential reasons to why the calcium hydroxide-related products that include MTA are generally believed to be the best choice for pulp capping [28].

Calcium hydroxide or MTA, when placed on an exposed pulp tissue, forms a necrotic layer due to high pH. The adjacent pulp tissue is responsible for pulp repair and dentin bridge formation [6]. The cytotoxicity of the tested materials, which was observed in this study, is causing the necrotic layer when the materials are in direct contact with pulp tissue. Unfortunately, the process of pulp tissue regeneration cannot be observed in the *in vitro* model. Considering the *in vivo* and clinical observations of the different pulp capping materials, calcium hydroxide has been the first choice for direct pulp capping [6]. MTA also has the comparable outcome [5]. This study suggests that cytotoxicity levels of pulp capping materials may not be the indication of their clinical success. Nevertheless, the low cytotoxicity of SB-C&B suggests its potential use in clinical investigations in future.

## Figures and Tables

**Table 1:** Manufacturer, working times, and setting times of pulp capping materials.

Materials	Manufacturer	Working time (min)	Setting time (min)
Dycal	Dentsply	2.5	2.0–3.0
Life	Kerr	>6.0	<7
MTA	Dentsply Tulsa	5.0–15.0	240.0–360.0
SB-C&B	Sun Medical	1.0–2.0	5.0–6.0

## References

[1] Pashley DH (1996). Dynamics of the pulpo-dentin complex. *Critical Reviews in Oral Biology and Medicine*.

[2] Bergenholtz G (2005). Advances since the paper by Zander and Glass (1949) on the pursuit of healing methods for pulpal exposures: historical perspectives. *Oral Surgery, Oral Medicine, Oral Pathology, Oral Radiology and Endodontology*.

[3] Schröder U (1985). Effects of calcium hydroxide-containing pulp-capping agents on pulp cell migration, proliferation, and differentiation. *Journal of Dental Research*.

[4] Cvek M (1978). A clinical report on partial pulpotomy and capping with calcium hydroxide in permanent incisors with complicated crown fracture. *Journal of Endodontics*.

[5] Mente J, Geletneky B, Ohle M (2010). Mineral trioxide aggregate or calcium hydroxide direct pulp capping: an analysis of the clinical treatment outcome. *Journal of Endodontics*.

[6] Hörsted-Bindslev P, Vilkinis V, Sidlauskas A (2003). Direct capping of human pulps with a dentin bonding system or with calcium hydroxide cement. *Oral Surgery, Oral Medicine, Oral Pathology, Oral Radiology, and Endodontics*.

[7] Hermann B (1930). Dentinobliteration der wurzelkanäle nach behandlung mit calzium. *Zahnärztl Rundschau*.

[8] Matsuo T, Nakanishi T, Shimizu H, Ebisu S (1996). A clinical study of direct pulp capping applied to carious-exposed pulps. *Journal of Endodontics*.

[9] Hilton TJ (2009). Keys to clinical success with pulp capping: a review of the literature. *Operative Dentistry*.

[10] Roberts HW, Toth JM, Berzins DW, Charlton DG (2008). Mineral trioxide aggregate material use in endodontic treatment: a review of the literature. *Dental Materials*.

[11] Camilleri J, Pitt Ford TR (2006). Mineral trioxide aggregate: a review of the constituents and biological properties of the material. *International Endodontic Journal*.

[12] Cox CF, Hafez AA, Akimoto N, Otsuki M, Suzuki S, Tarim B (1998). Biocompatibility of primer adhesive and resin composite systems on non-exposed and exposed pulps of non-human primate teeth. *American Journal of Dentistry*.

[13] de Lourdes Rodrigues Accorinte M, Loguercio AD, Reis A, Muench A, de Araújo VC (2005). Adverse effects of human pulps after direct pulp capping with the different components from a total-etch, three-step adhesive system. *Dental Materials*.

[14] Imaizumi N, Kondo H, Ohya K, Kasugai S, Araki K, Kurosaki N (2006). Effects of exposure to 4-META/MMA-TBB resin on pulp cell viability. *Journal of Medical and Dental Sciences*.

[15] Nakamura M, Inoue T, Shimono M (2000). Immunohistochemical study of dental pulp applied with 4-META/MMA-TBB adhesive resin after pulpotomy. *Journal of Biomedical Materials Research*.

[16] Kato C, Suzuki M, Shinkai K, Katoh Y (2011). Histopathological and immunohistochemical study on the effects of a direct pulp capping experimentally developed adhesive resin system containing reparative dentin-promoting agents. *Dental Materials Journal*.

[17] Pissiotis E, Spångberg LS (1991). Toxicity of Pulpispad using four different cell types. *International Endodontic Journal*.

[18] Cvek M (1978). A clinical report on partial pulpotomy and capping with calcium hydroxide in permanent incisors with complicated crown fracture. *Journal of Endodontics*.

[19] Hebling J, Giro EMA, de Souza Costa CA (1999). Biocompatibility of an adhesive system applied to exposed human dental pulp. *Journal of Endodontics*.

[20] de Souza Costa CA, Lopes do Nascimento AB, Teixeira HM, Fontana UF (2001). Response of human pulps capped with a self-etching adhesive system. *Dental Materials*.

[21] Hirschman WR, Wheater MA, Bringas JS, Hoen MM (2012). Cytotoxicity comparison of three current direct pulp-capping agents with a new bioceramic root repair putty. *Journal of Endodontics*.

[22] Tani-Ishii N, Hamada N, Watanabe K, Tujimoto Y, Teranaka T, Umemoto T (2007). Expression of bone extracellular matrix proteins on osteoblast cells in the presence of mineral trioxide. *Journal of Endodontics*.

[23] Mozayeni MA, Milani AS, Marvasti LA, Asgary S (2012). Cytotoxicity of calcium enriched mixture cement compared with mineral trioxide aggregate and intermediate restorative material. *Australian Endodontic Journal*.

[24] Yasuda Y, Ogawa M, Arakawa T, Kadowaki T, Saito T (2008). The effect of mineral trioxide aggregate on the mineralization ability of rat dental pulp cells: an *in vitro* study. *Journal of Endodontics*.

[25] Tronstad L, Spangberg L (1974). Biologic tests of a methyl methacrylate composite material. *Scandinavian Journal of Dental Research*.

[26] Yoshii E (1997). Cytotoxic effects of acrylates and methacrylates: relationships of monomer structures and cytotoxicity. *Journal of Biomedical Materials Research*.

[27] Fridland M, Rosado R (2005). MTA solubility: a long term study. *Journal of Endodontics*.

[28] Modena KC, Casas-Apayco LC, Atta MT (2009). Cytotoxicity and biocompatibility of direct and indirect pulp capping materials. *Journal of Applied Oral Science*.

